# Membrane Depolarization and Apoptosis-Like Cell Death in an Alkaline Environment in the Rice Pathogen *Burkholderia glumae*

**DOI:** 10.3389/fmicb.2021.755596

**Published:** 2021-10-12

**Authors:** Yewon Nam, Eunhye Goo, Yongsung Kang, Ingyu Hwang

**Affiliations:** ^1^Department of Agricultural Biotechnology, Seoul National University, Seoul, South Korea; ^2^Research Institute of Agriculture and Life Sciences, Seoul National University, Seoul, South Korea

**Keywords:** apoptosis-like cell death, membrane depolarization, cellular pH, *Burkholderia glumae*, rice panicle blight

## Abstract

The rice pathogen *Burkholderia glumae* uses amino acids as a principal carbon source and thus produces ammonia in amino acid-rich culture medium such as Luria–Bertani (LB) broth. To counteract ammonia-mediated environmental alkaline toxicity, the bacterium produces a public good, oxalate, in a quorum sensing (QS)-dependent manner. QS mutants of *B. glumae* experience alkaline toxicity and may undergo cell death at the stationary phase when grown in LB medium. Here, we show that the cell-death processes of QS mutants due to alkaline environmental conditions are similar to the apoptosis-like cell death reported in other bacteria. Staining QS mutants with bis-(1,3-dibutylbarbituric acid)-trimethine oxonol revealed membrane depolarization. CellROX™ staining showed excessive generation of reactive oxygen species (ROS) in QS mutants. The expression of genes encoding HNH endonuclease (BGLU_1G15690), oligoribonuclease (BGLU_1G09120), ribonuclease E (BGLU_1G09400), and Hu-beta (BGLU_1G13530) was significantly elevated in QS mutants compared to that in wild-type BGR1, consistent with the degradation of cellular materials as observed under transmission electron microscopy (TEM). A homeostatic neutral pH was not attainable by QS mutants grown in LB broth or by wild-type BGR1 grown in an artificially amended alkaline environment. At an artificially adjusted alkaline pH, wild-type BGR1 underwent apoptosis-like cell death similar to that observed in QS mutants. These results show that environmental alkaline stress interferes with homeostatic neutral cellular pH, induces membrane depolarization, and causes apoptosis-like cell death in *B. glumae*.

## Introduction

Bacteria have survival mechanisms to adapt to stressful conditions deviating from their niche conditions. The ecological systems in which bacteria live are diverse, with varying temperatures, nutrients, osmolarity, and pH. To cope with a stressful environment, bacteria use specific molecular and metabolic mechanisms. For example, bacteria produce heat shock proteins and can form endospores in response to heat stress (Yura et al., 1993; Russell, 2003). When nutrients are limited, bacterial cells reduce their size and increase the expression of specific genes to maintain continuous growth and survival (Ishihama, 1997; Nyström, 2004). In a highly osmotic external environment, bacteria possess several strategies for adjusting their cellular osmolarity through the import of ions and the synthesis of compatible solutes (Imhoff, 1986; Wood, 2006). Because pH is a powerful stress factor that can affect a variety of biological molecules, bacteria must maintain a cellular pH in the neutral range. How *Escherichia coli* maintains a neutral cellular pH in response to pH fluctuations during growth has been well studied (Slonczewski et al., 1981; Taglicht et al., 1993; Maurer et al., 2005). The survival mechanisms of bacteria in stressful environments are also well understood to date (Aertsen and Michiels, 2004; Justice et al., 2008). However, the molecular and physiological details of bacterial cell death under non-permissible conditions are less characterized.

When bacteria encounter stressful environments or antibacterial agents, adaptation is necessary for survival. The identification of primary targets of antibacterial agents is an important first step in improving our understanding of how bacterial cells die. However, more effort is needed to elucidate the molecular biological or physiological processes that kill bacteria under insurmountable environmental conditions. Death of *E. coli* upon exposure to antibacterial agents mimics apoptosis, also known as programmed cell death, in multicellular organisms (Sat et al., 2001; Dwyer et al., 2012). Apoptosis-like cell death is additionally caused by oxidative stress and UV radiation and appears to be a common phenomenon in bacteria (Chandra et al., 2000; Hazan et al., 2004). Recent studies have shown that a common downstream cellular event following exposure to antibacterial agents is the production of reactive oxygen species (ROS), which are considered a potent inducer of apoptosis-like cell death (Chandra et al., 2000; Dwyer et al., 2012). In fact, several bactericidal antibiotics have been shown to induce the generation of intracellular ROS followed by DNA damage and thus cause apoptosis-like cell death (Dwyer et al., 2012, 2014). However, it is not known whether bacterial cell death caused by other factors such as pH stress is similar to apoptosis-like cell death.

We investigated how the rice pathogen *Burkholderia glumae* BGR1 survives under harsh environmental conditions. This bacterium causes bacterial panicle blight in rice through flower infection and has been shown to use the amino acids in Luria–Bertani (LB) medium as its major carbon source (Goo et al., 2012). The LuxI-R-type quorum sensing (QS) system, TofI-R, is responsible for the generation of *N*-octanoyl homoserine lactone (C8-HSL), the major QS signaling molecule in *B. glumae* (Kim et al., 2004). The TofR and C8-HSL complex activates the expression of *qsmR* encoding an isocitrate lyase regulator (IclR)-type transcriptional regulator (Kim et al., 2007). qsmR in turn activates the expression of *obcAB* encoding enzymes for oxalate biosynthesis in *B. glumae* (Kim et al., 2007; Goo et al., 2012). When *B. glumae* is grown in LB medium, it uses amino acids as its major carbon source and releases ammonia into the culture as a result of amino-acid deamination (Goo et al., 2012, 2015). Such ammonia-mediated alkaline environmental conditions were found to be only toxic to QS mutants of *B. glumae* because the wild-type BGR1 produced a public good, oxalate, in a QS-dependent manner to counteract alkaline toxicity (Goo et al., 2012). These results led us to investigate the molecular and physiological mechanisms involved in cell death in QS mutants at alkaline pH. We first aimed to determine whether cell death in QS mutants was similar to the known apoptosis-like cell death in other bacteria. Second, we aimed to determine whether wild-type BGR1 was sensitive to alkaline environmental pH. Finally, we compared the molecular mechanisms involved in cell death in wild-type BGR1 at alkaline pH with those of QS mutants in LB medium. We found that the QS mutants underwent membrane depolarization and apoptosis-like cell death as in other bacteria. Our results showed that wild-type BGR1 was sensitive to alkaline environmental pH; we then investigated cell death in the QS mutants. Our findings revealed that *B. glumae* is inherently sensitive to alkaline environmental pH, and the mechanism underlying alkaline stress-induced cell death is similar to that by which apoptosis-like cell death occurs in other bacteria.

## Materials and Methods

### Bacterial Strains and Growth Conditions

The bacterial strains and plasmids used in this study are listed in Supplementary Table S1. All *B. glumae* and *E. coli* strains were aerobically grown in LB broth [0.1% tryptone, 0.5% yeast extract, and 0.5% NaCl (w/v); USB, Cleveland, OH, United States] or in LB broth buffered with 100mM 4-(2-hydroxyethyl) piperazin-1 ethanesulfonic acid (HEPES; Sigma-Aldrich, St. Louis, MO, United States) to maintain external pH at pH 7 at 37°C. When necessary, the LB broth was supplemented with 1μM C8-HSL, 10μg/ml gentamicin, and 50μM nigericin for growth experiments. For the alkaline-stress experiments, the wild-type *B. glumae* BGR1 and *E. coli* DH5α strains were diluted to an optical density (OD) of 0.05 based on absorbance at 600nm (A_600_) and grown overnight for subculture. The cultures were washed twice with LB broth and transferred to LB broth buffered with 100mMN-(1,1-dimethyl-2-hydroxyethyl)-3-amino-2-hydroxypropanesulfonic acid (AMPSO) at pH 8, 9, or 10 (Sigma-Aldrich).

### Transmission Electron Microscopy

Wild-type *B. glumae* BGR1 and the QS mutants BGS2 (BGR1 *tofI*::Ω) and BGS9 (BGR1 *qsmR*::Ω) were grown at 37°C for 20, 24, and 28h in LB broth. To induce alkaline stress, wild-type BGR1 was transferred to LB broth buffered with AMPSO (pH 9) for 1, 4, and 8h. These cells were then harvested *via* centrifugation at 10,000×*g* for 10min and examined using transmission electron microscopy (TEM) as previously described (Kang and Hwang, 2018). Electron micrographs were acquired using a LIBRA 120 energy-filtration microscope (Carl Zeiss, Oberkochen, Germany) at 100kV.

### Fluorescence Staining

*Burkholderia glumae* and *E. coli* DH5α cells were collected and stained with the following fluorescent dyes: LIVE/DEAD BacLight™, 40,6-diamidino-2-phenylindole dihydrochloride (DAPI), SYTO™ RNASelect™ Green Fluorescent cell stain, and CellROX™ Deep Red Reagent.

The numbers of live and dead bacteria were determined using a LIVE/DEAD BacLight™ bacterial viability kit (Life Technologies, Carlsbad, CA, United States). The cells were stained with a mixture of SYTO9 and propidium iodide (1:1) for 20min in the dark at room temperature (RT). DNA was stained with DAPI (Life Technologies) at a final concentration of 20ng/ml in the dark for 20min at RT, and RNA was visualized using the SYTO™ RNASelect™ Green Fluorescent cell stain (Life Technologies) at a final concentration 500nM in the dark for 20min at 37°C. ROS levels were quantified after staining with CellROX™ Deep Red Reagent (Life Technologies) as previously described with some modifications (Grinberg et al., 2012, 2013). CellROX™ reagent at a final concentration of 5μM was added to *B. glumae* or *E. coli* DH5α, and the cells were incubated in the dark for 30min at 37°C. All stained cells were washed twice and resuspended in phosphate-buffered saline (PBS), with the exception of cells stained with SYTO™ RNASelect™, which were washed with LB broth. Fluorescence images were immediately obtained using an Olympus BX53 fluorescent microscope (Olympus, Tokyo, Japan).

### Membrane Depolarization Assay

To examine membrane disturbance, *B. glumae* and *E. coli* DH5α cells were harvested *via* centrifugation and resuspended in PBS. Subsequently, the cells were treated with 5μM 3,3′-dipropylthiadicarbocyanine iodide [DiSC_3_(5); Life Technologies] and 19μM bis-(1,3-dibutylbarbituric acid) trimethine oxonol [DiBAC_4_(3); Life Technologies] for 20min in the dark at RT. DiSC_3_(5) levels were measured using a SpectraMax M2e microplate reader (Molecular Devices, San Jose, CA, United States), and DiBAC_4_(3) signals were observed under an Olympus BX53 fluorescent microscope (Olympus). In addition, 50μM nigericin was added to wild-type BGR1 to induce factitious membrane depolarization.

### Quantitative Reverse-Transcription PCR

An RNeasy Mini kit (Qiagen, Venlo, Netherland) was used to isolate total RNA from wild-type *B. glumae* BGR1 and QS mutants in LB broth cultured at 37°C for 10h. Total RNA was isolated from wild-type BGR1 after 4h of alkaline stress at pH 9 or at 8h after the addition of 50μM nigericin. RNase-free DNaseI treatment of the isolated RNA for 30min at 37°C, reverse transcription to cDNA at 42°C for 1h, and reverse-transcription PCR (RT-PCR) were performed as described previously (Jang et al., 2014). The primer pairs used for quantitative reverse-transcription PCR (qRT-PCR) are listed in Supplementary Table S2. qRT-PCR analysis was performed to compare transcriptional levels using SsoFast EvaGreen Supermix (Bio-Rad, Hercules, CA, United States) and the CFX96 Real-Time PCR system (Bio-Rad). The thermal cycling parameters were as follows: 95°C for 30s, followed by 35cycles of 95°C for 5s and 60°C for 5s. All reactions were run in triplicate from three independent cultures with three technical repeats per dose, and the 16S rRNA gene was used for data normalization.

### Quantification of DNA Damage

For the quantification of DNA damage, we evaluated the levels of damage to DNA gyrase A (BGLU_1G08730) relative to that in undamaged controls as described previously (Patenge, 2017). Amplifications were normalized by DNA gyrase A copy number by concurrently amplifying a “short-range” PCR fragment. For the DNA gyrase A product, we selected the following primer sequences: long forward, 5′-CGAACTGAACAACGACTGGA-3′; short forward, 5′-AGCTGCAGGACACGTTCG-3′; and long and short reverse, 5′-GCATCTCCTTCAGGTTCAGC-3′. The lesion frequency per amplicon was calculated assuming a random distribution of lesions as described previously (Patenge, 2017).

### Green Fluorescent Protein Fusion and Measurement of GFP Fluorescence Intensity

To determine the protein expression levels in wild-type BGR1 and QS mutants, GFP-tagged Hu-beta and green fluorescent protein (GFP)-tagged HNH endonuclease were constructed as described previously (Jang et al., 2014). Hu-beta and HNH endonuclease genes with the promoter region and lacking the stop codon were cloned into pJW23 containing an enhanced GFP (eGFP) gene and subcloned into pUC18R6KT miniTn7T-Tc. Subsequently, Mini-Tn7 expressing Hu-beta and HNH endonuclease with eGFP was integrated into the *glmS* gene of *B. glumae* (Choi et al., 2006). GFP fluorescence was measured using a PerkinElmer Victor X3 microplate reader (PerkinElmer, Waltham, MA, United States).

### Intracellular pH Determination

To measure the internal pH, we constructed the plasmid pPHI4 containing the *trc* promoter and ratiometric pHluorin. For plasmid construction, the ratiometric pHluorin gene (GenBank accession no. AF058694.2) was synthesized. Overnight bacterial cultures were aerobically grown in LB broth at 37°C and diluted to an OD of 0.05 based on A_600_. After 14h of subculture, bacteria in 2ml of each culture were harvested *via* centrifugation (10,000×*g* for 2min), washed twice with 1ml of Dulbecco’s phosphate-buffered saline (DPBS; 150mM NaCl, 3mM KCl, 1mM KH_2_PO_4_, 6mM Na_2_HPO_4_, 0.5mM MgCl_2_, and 1mM CaCl_2_) as previously described with some modifications (Reifenrath and Boles, 2018), and resuspended in 1ml of DPBS. To induce alkaline stress, wild-type *B. glumae* BGR1 and *E. coli* DH5α were placed in DPBS at pH 9.

To construct a calibration curve to measure the internal pH in all strains, harvested cells were equilibrated in DPBS adjusted to different pH values (pH 5, 6, 7, 8, and 9) in the presence of 40mM potassium benzoate and 40mM methylamine hydrochloride before detection of fluorescence intensity (Martinez et al., 2012; Reyes-Fernández and Schuldiner, 2020). Benzoic acid (permeant acid) and methylamine hydrochloride (permeant base) were used to collapse the pH difference across the membrane and equialize the external and internal pH. Aliquots of 200μl of each cell suspension were distributed into a 96-well plate, and the fluorescence produced was measured by scanning a range of excitation wavelengths (330–482nm) with emission at 510nm using a SpectraMax M2e microplate reader (Molecular Devices). Because the excitation peaks of dual wavelength in *B. glumae* and *E. coli* DH5a were different, the 370/470-nm fluorescence excitation ratios (R_370/470_) of *B. glumae* and R_410/470_ values of *E. coli* DH5α were determined, respectively. Finally, the calibration curves were constructed using designated excitation ratio, R_370/470_ or R_410/470_, and the internal pH was calculated as described previously (Martinez et al., 2012; Crauwels et al., 2018; Arce-Rodríguez et al., 2019; Reyes-Fernández and Schuldiner, 2020).

### Expression of Sodium–Proton Antiporters in *B. glumae*

DNA cloning, restriction mapping, and gel electrophoresis were used to introduce *nhaA* into *E. coli* (Sambrook et al., 1989). We constructed pNha13, in which the native promoter and *nhaA* of *E. coli* (GenBank accession no. YP_001729002.1) were expressed. For plasmid construction, the coding region of the native promoter and *nhaA* was amplified with the primers NhaA_F (5′-CCAAGAGCTCCTATCTGCCGTTCAGCTAATGC-3′) and NhaA_R (5′-ACCGTGGGCCCCGTGTCA-3′′) using the genomic DNA of *E. coli* DH5α. The amplified PCR product containing *Sac*I and *Kpn*I recognition sites was inserted into pLAFR6, resulting in pNha13 (Supplementary Tables S1, S2).

To test the constitutive expression of genes encoding putative Na^+^/H^+^ antiporter genes from *B. glumae*, the promoter region was replaced with the *trc* promoter. Genes annotated as related to the Na^+^/H^+^ antiporter of *B. glumae* were amplified with the primers listed in Supplementary Table S2. The amplified PCR product was digested with *Nde*I and *Bam*HI and inserted into pBP1 such that the translation initiation codon of the putative Na^+^/H^+^ antiporter gene was fused to the *trc* promoter. The DNA fragments carrying the putative Na^+^/H^+^ antiporter genes (BGLU_1G32530, 1G09320, 2G00450, and 2G17030) with *trc* promoter obtained from pNha4, pNha5, pNha22, and pNha23 *via* digestion performed with *Sac*I and *Kpn*I, *Sac*I and *Bam*HI, or *Xba*I and *Hind*III, were cloned into pLAFR6 to produce pNha7, pNha8, pNha24, and pNha25. Furthermore, pLAFR6 containing *E. coli nhaA* with *trc* promoter was designated pTrc-nhaA (Supplementary Table S1).

### Statistical Analysis

All statistical analyses and ANOVA testing followed by Tukey’s honest significance difference *post hoc* analysis were performed using IBM SPSS Statistics software (ver. 20 x86-x64; IBM Corp., Armonk, NY, United States).

## Results

### QS Mutants Undergo Apoptosis-Like Cell Death Based on Cell Biological Evidence

**Figure 1:**
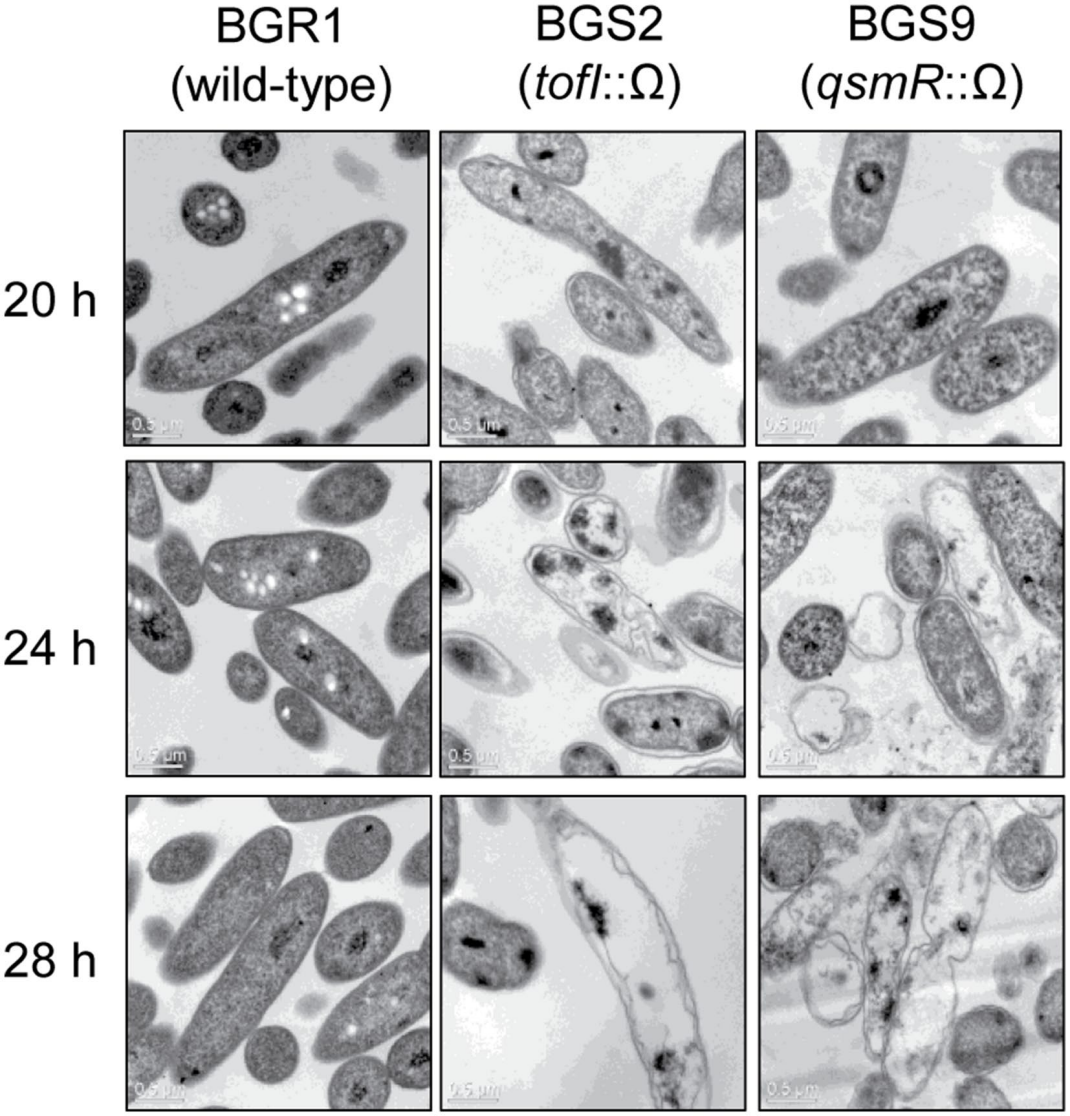
Transmission electron microscopy (TEM) images of wild-type BGR1 and the quorum sensing (QS) mutants BGS2 (BGR1 *tofI*::Ω) and BGS9 (BGR1 *qsmR*::Ω). Wild-type BGR1 and the QS mutants were grown at 37°C in Luria–Bertani (LB) broth with shaking. Scale bars, 0.5μm.

To determine whether cell death in QS mutants at alkaline environmental pH is similar to the known apoptosis-like cell death in other bacteria, we first observed the cellular contents of two QS mutants, BGS2 (BGR1 *tofI*::Ω) and BGS9 (BGR1 *qsmR*::Ω), using TEM during the stationary phase. TEM images showed that the cellular materials of the QS mutants were less negatively stained with uranyl acetate than those of wild-type BGR1 (Figure 1). Uranyl acetate, which produces negative stain contrast by interaction with lipids, proteins, and nucleic acid phosphate groups, was used as a stain and fixative for electron microscopy. Cytoplasmic membranes were intact at the initial stage of cell death in the QS mutants; these mutants appeared as ghost-like cells at 24h after incubation in LB broth at 37°C (Figure 1).

**Figure 2:**
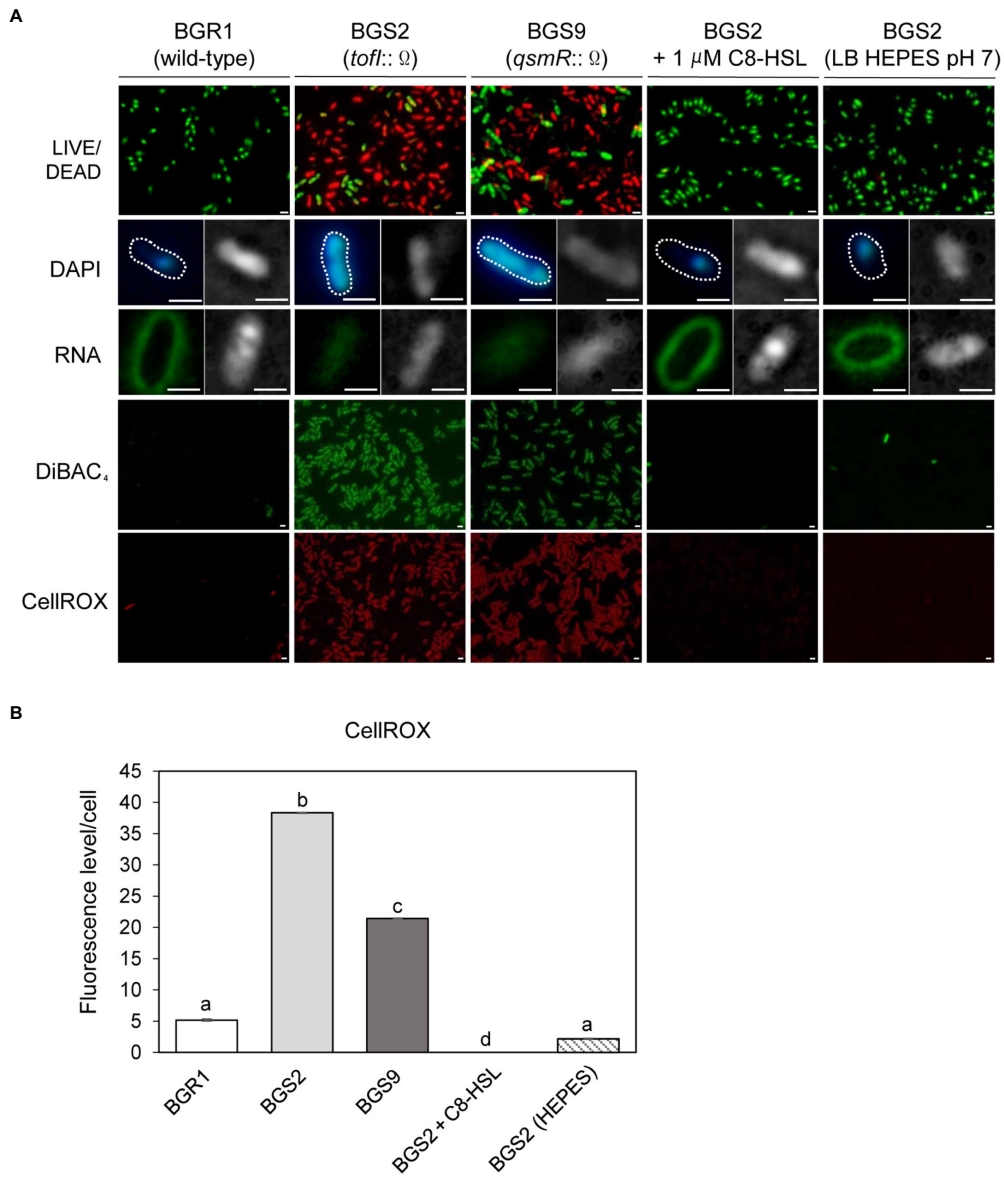
Fluorescence staining of wild-type BGR1 and QS mutants after 24h in LB broth at 37°C. **(A)** Cell viability was assayed *via* LIVE/DEAD BacLight™ cell staining. Live and dead cells were detected as green and red fluorescence, respectively. DNA was stained with 40,6-diamidino-2-phenylindole dihydrochloride (DAPI) and RNA was stained with SYTO™ RNASelect™ (Rows 2 and 3). Differential interference contrast (DIC) microscopy (right) images and merged DIC/fluorescence images (left) with DAPI or SYTO™ RNASelect™ staining are depicted. Wild-type BGR1 and the QS mutants were treated with DiBAC_4_(3) to observe membrane depolarization and CellROX to measure cellular reactive oxygen species (ROS) levels. BGS2 (BGR1 *tofI*::Ω) cells were supplemented with 1μM of C8-HSL and 100mM 4-(2-hydroxyethyl) piperazin-1 ethanesulfonic acid (HEPES; pH 7). Scale bars, 1μm. **(B)** Quantitative imaging analysis of ROS levels based on CellROX staining intensity. The fluorescence intensity was analyzed using ImageJ version 1.53a software (NIH). Data are mean±SE of triplicate experiments. The letters (a, b, c, and d) above each mean represent groupings of statistical significance based on ANOVA/Tukey’s correction for multiple comparisons. A value of *p*<0.05 represents significant differences among strains.

To further highlight similarities between cell death in the QS mutants and the known apoptosis-like cell death in other bacteria, we employed cell biological tools. Live and dead cell staining with the LIVE/DEAD BacLight™ kit revealed that the vast majority of QS mutant cells were dead (red) after 24h (Figure 2A). DAPI staining showed that DNA in the QS mutants was disintegrated, unlike the condensed forms observed in wild-type BGR1, and RNASelect™ staining indicated that RNA appeared to be degraded in the QS mutants compared to wild-type BGR1 based on fluorescence intensity (Figure 2A). Furthermore, membrane depolarization was observed in the QS mutants upon DiBAC_4_(3) staining (Figure 2A). Excessive generation of ROS was detected in the QS mutants when cells in the stationary phase were stained with CellROX™ Deep Red Reagent (Figures 2A,B). However, when BGS2 (BGR1 *tofI*::Ω) was cultured in LB broth supplemented with 1μM C8-HSL or 100mM HEPES (pH 7), all cell-death phenotypes disappeared, and the phenotype was restored to that of wild-type BGR1 (Figure 2).

**Figure 3:**
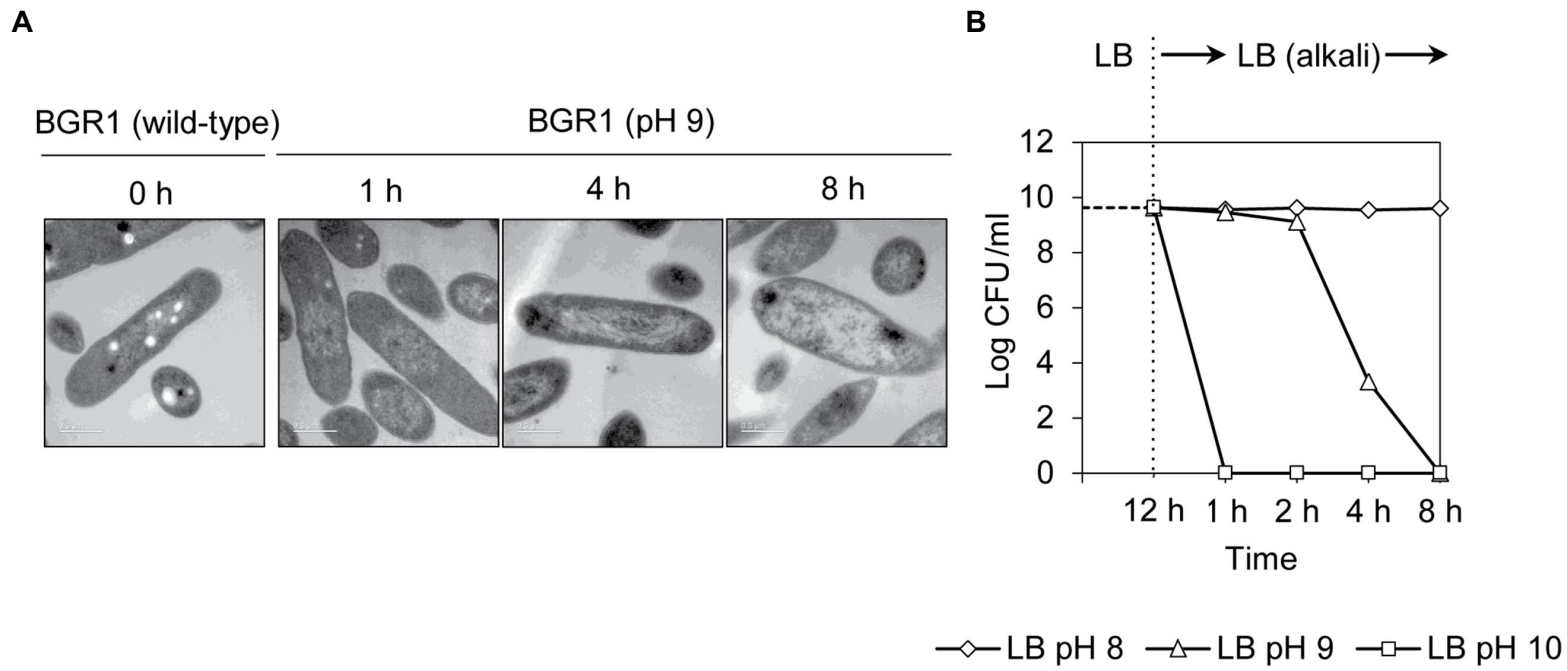
Cell death in wild-type BGR1 induced by alkaline stress. **(A)** TEM images of wild-type BGR1 after exposure to pH 9 at different times. Scale bars, 0.5μm. **(B)** Cell viability of wild-type BGR1 following exposure to alkaline stress. Wild-type BGR1 was artificially challenged at pH 8, 9, or 10 to induce alkaline stress at 37°C. The cell population was determined based on the number of colony-forming units (CFUs) through plate-counting methods.

**Figure 4:**
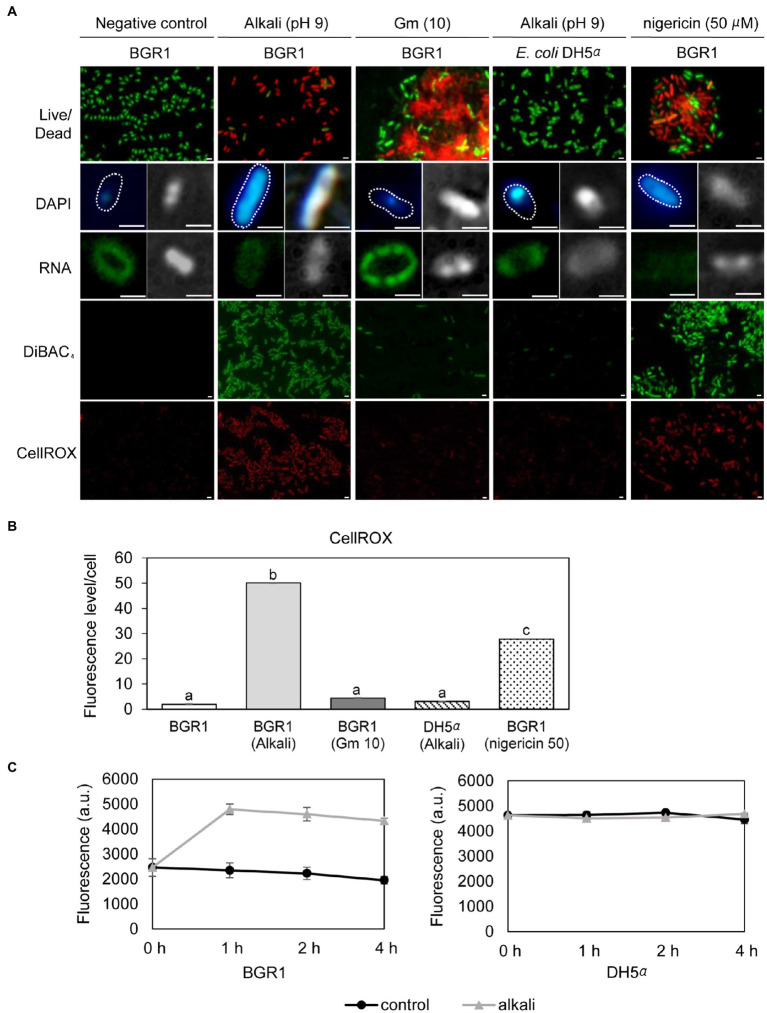
Fluorescence staining of wild-type BGR1 and *Escherichia coli* DH5α after 8h of alkaline stress at pH 9 or antibiotic treatment with shaking at 37°C. **(A)** LIVE/DEAD, DAPI, and SYTO™ RNASelect™ staining were performed after 8h of alkaline stress at pH 9. For DAPI and SYTO™ RNASelect™ staining, DIC microscopy (right) and merged DIC/fluorescence (left) images are depicted. Cells were stained with DiBAC_4_(3) and CellROX to observe membrane depolarization and FIGURE 4 | ROS, respectively. Gentamicin [10μg/ml, indicated by Gm (10)] and 50μM nigericin were added to wild-type BGR1. Scale bars, 1μm. **(B)** Quantitative imaging analysis of ROS levels based on CellROX staining intensity. The fluorescence intensity was analyzed using ImageJ software. The letters (a, b, and c) above each mean represent groupings of statistical significance based on ANOVA/Tukey’s correction for multiple comparisons. A value of *p*<0.05 represents significant differences among strains. **(C)** The fluorescence intensity of the membrane charge-sensitive dye DiSC_3_(5) was monitored at excitation/emission wavelengths of 651/675nm using a SpectraMax M2e microplate reader. Data are mean±SE of triplicate experiments.

Because cell death in the QS mutants was due to the alkaline environmental pH, we examined whether artificially amended alkaline conditions induced the same phenomenon in wild-type BGR1. The phenotype of wild-type BGR1 exposed to pH 9 was similar to that of dead QS mutants in the stationary phase in the TEM images (Figure 3A); consequently, these cells exhibited a decreased population density (Figure 3B). Cell biological assays of wild-type BGR1 challenged with alkaline stress also produced results similar to those obtained for the QS mutants during the stationary phase, suggesting DNA disintegration and RNA degradation (Figure 4A). The occurrence rates of ROS and membrane depolarization in wild-type BGR1 were higher under alkaline stress than under normal conditions (Figures 4A,B). Monitoring of the membrane charge-sensitive dye DiSC_3_(5) verified the occurrence of membrane depolarization in wild-type BGR1 under alkaline stress (Figure 4C). *Escherichia coli* DH5α cells exposed to pH 9 did not exhibit an apoptosis phenotype (Figure 4). Moreover, wild-type BGR1 treated with gentamicin, a bacterial antibiotic that binds the 30S subunit of the bacterial ribosome and disturbs protein synthesis by misreading mRNA, did not exhibit an apoptosis-like cell-death phenotype despite dead cells being observed with LIVE/DEAD staining (Figures 4A,B). These results suggest that the cell death mechanism induced by alkaline stress in *B. glumae* was different than that attributable to gentamicin.

### Upregulation of Genes Encoding Nucleotide-Degradation Enzymes

**Figure 5:**
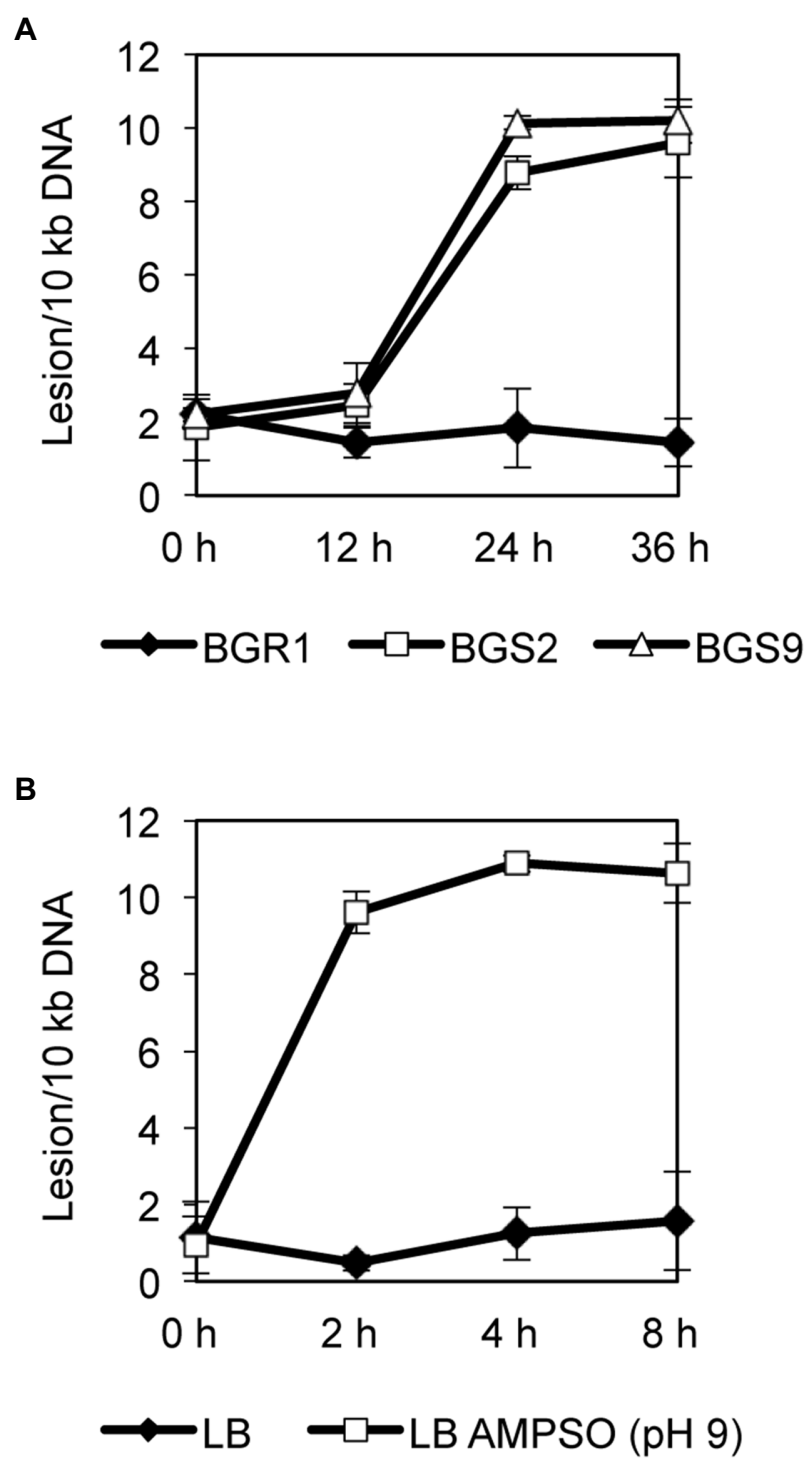
Quantitative analysis of nucleic acid damage. **(A)** Quantitative PCR assay for DNA damage in wild-type BGR1 and QS mutants in LB broth at 37°C. The relative levels of DNA damage were evaluated by comparing the lesion frequency per amplicon between wild-type BGR1 and QS mutants. **(B)** Quantitative PCR assay for DNA damage in wild-type BGR1 after transfer to LB broth or LB broth buffered with 100mM AMPSO (pH 9). Data are mean±SE of triplicate experiments.

To observe whether DNA damage similar to that reported in apoptosis-like cell death in other bacteria occurred during cell death in QS mutants, we evaluated the extent of DNA damage in wild-type BGR1 and the QS mutants BGS2 (BGR1 *tofI*::Ω) and BGS9 (BGR1 *qsmR*::Ω) using qRT-PCR. Quantitative analysis of nucleic acid damage in wild-type BGR1 and the QS mutants showed lesion rates suggesting fivefold higher damage per 10kb DNA in the QS mutants than in wild-type BGR1 after 24h (Figure 5A). We also showed that artificially adjusted alkaline conditions caused increased DNA damage, fivefold that observed in wild-type BGR1 under non-alkaline conditions (Figure 5B).

**Figure 6:**
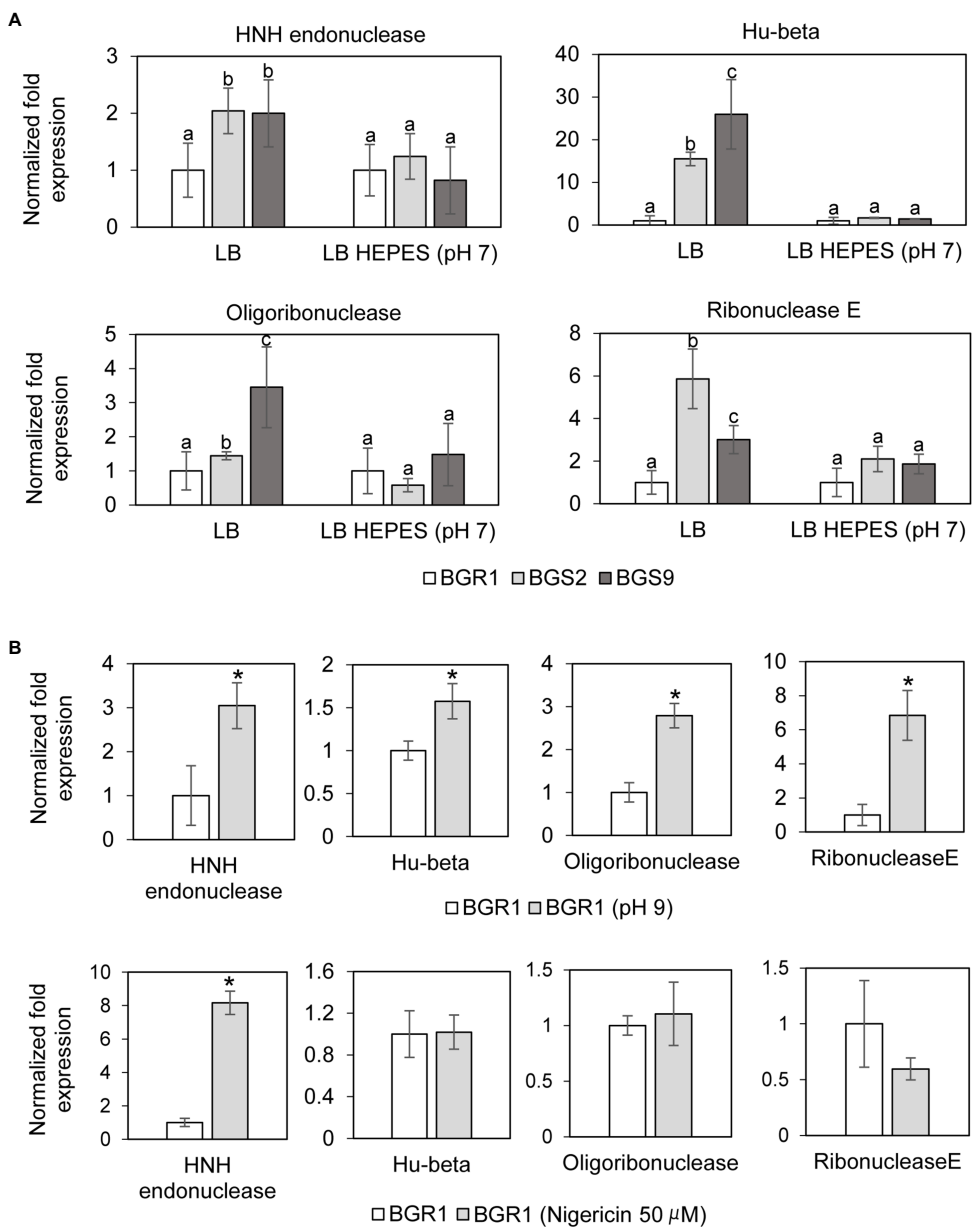
Relative expression of genes encoding nucleotide-degrading enzymes. **(A)** The expression levels of genes encoding HNH endonuclease, Hu-beta, oligoribonuclease, and ribonuclease E were compared between wild-type BGR1 and QS mutants in LB broth or HEPES-buffered LB broth (pH 7) after 10h, as revealed by quantitative reverse-transcription PCR (qRT-PCR). Data are mean±SE of triplicate experiments. The letters (a, b, and c) above each mean represent groupings of statistical significance based on ANOVA/Tukey’s correction for multiple comparisons. A value of *p*<0.05 represents significant differences among strains. **(B)** The relative expression of target genes in wild-type BGR1 was measured after 4h of alkaline stress at pH 9 or at 8h after the addition of 50μM nigericin. Data are mean±SE of triplicate experiments. The asterisks (*) indicate a significant difference in expression levels (*p*<0.05) compared to the wild type as determined by ANOVA/Tukey’s correction for multiple comparisons. The 16S rRNA gene was used for normalization in all PCR runs.

In addition to the roles of ROS in DNA damage, we also investigated whether nucleotide-degrading enzymes were involved in DNA damage in the QS mutants. Based on our previous RNA sequencing results from comparative studies on the gene expression profiles of wild-type BGR1 and the QS mutants (Supplementary Table S3; Goo et al., 2012), three genes encoding nucleic acid-degradation enzymes, HNH endonuclease (BGLU_1G15690), oligoribonuclease (BGLU_1G09120), and ribonuclease E (BGLU_1G09400), and a gene encoding the chromosomal binding protein Hu-beta (BGLU_1G13530) were chosen for further analysis. The qRT-PCR results showed that the expression of these genes was higher in the QS mutants than in wild-type BGR1 after 10h (Figure 6A). However, when both the QS mutants and wild-type BGR1 were grown in LB broth supplemented with 100mM HEPES (pH 7), the expression levels of the four genes were similar (Figure 6A). The increase in expression of the four genes was similarly reproduced in wild-type BGR1 exposed to alkaline stress (Figure 6B, upper panel). In addition, measurements of fluorescence intensity of GFP-tagged HNH endonuclease and Hu-beta signals confirmed that the expression of HNH endonuclease and Hu-beta was increased both in the QS mutants and wild-type BGR1 under alkaline stress (Supplementary Figure S1).

### Membrane Depolarization Is an Integral Part of Alkaline Stress-Induced Cell Death

To investigate whether membrane depolarization occurs during apoptosis-like cell death in QS mutants, we evaluated membrane depolarization in QS mutant and wild-type BGR1 cells undergoing apoptosis-like cell death following alkaline stress. When 50μM nigericin was added to wild-type BGR1 to induce membrane depolarization, the cell death patterns were similar to those observed with alkaline stress-induced cell death (Figures 4A,B). Addition of the membrane depolarization-inducing agent nigericin to wild-type BGR1 caused a significant increase in the expression of the HNH endonuclease gene compared to untreated wild-type BGR1 (Figure 6B, bottom panel). However, the expression of the genes encoding oligoribonuclease, ribonuclease E, and Hu-beta in wild-type BGR1 was not significantly affected by the addition of 50μM nigericin, unlike with alkaline stress (Figure 6B, bottom panel).

### Failure to Maintain pH Homeostasis Upon Exposure to Alkaline Conditions in *B. glumae*

**Figure 7:**
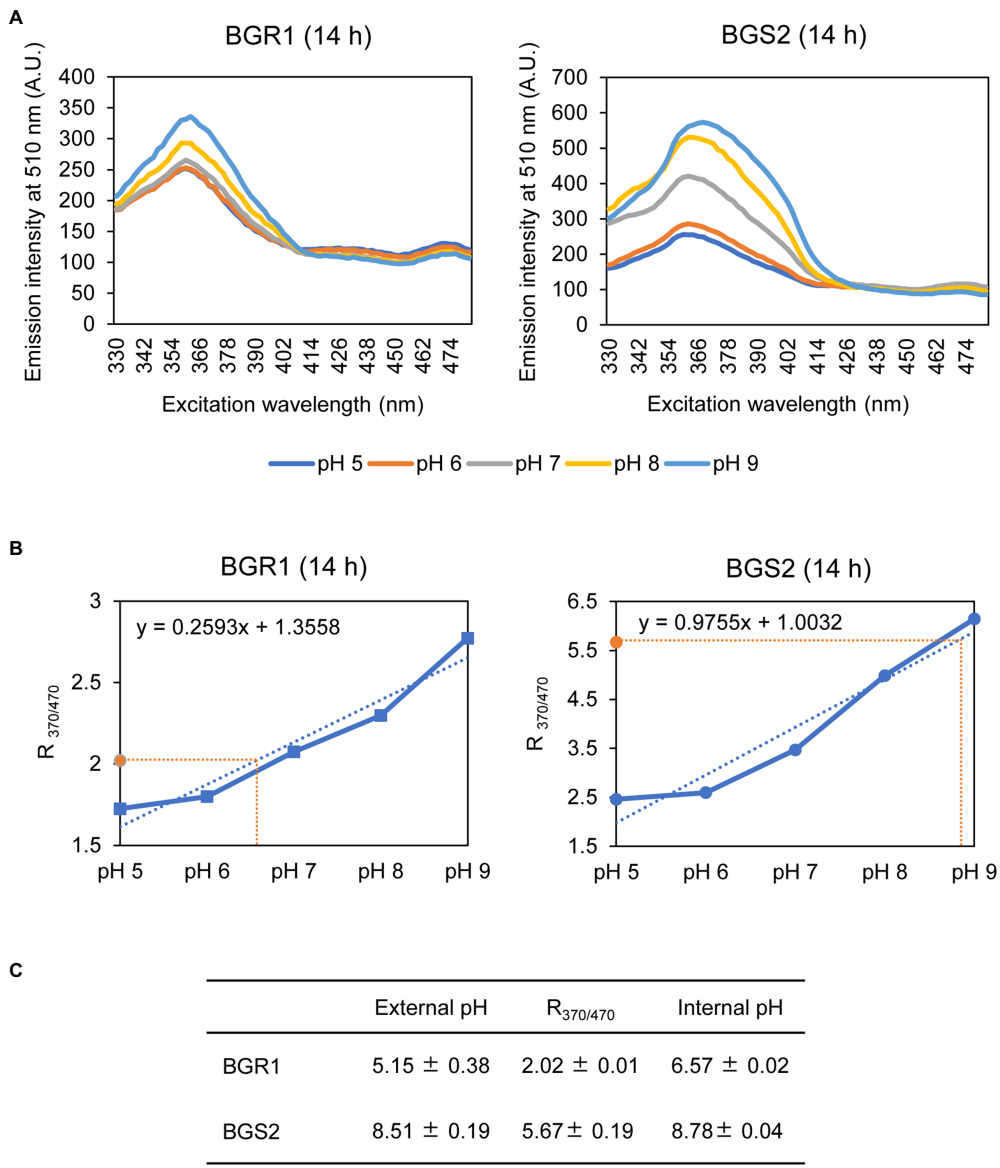
Measurement of the internal pH of wild-type BGR1 and BGS2 (BGR1 *tofI*::Ω). **(A)** Fluorescence excitation spectrum of the ratiometric fluorescent indicator pHluorin for wild-type BGR1 and BGS2 (330–482nm; emission, 510nm). Wild-type BGR1 and BGS2 (BGR1 *tofI*::Ω) were grown in LB broth for 14h at 37°C. Subsequently, the cells were resuspended in DPBS at different pH (pH 5, 6, 7, 8, or 9) in the presence of 40mM potassium benzoate and 40mM methylamine hydrochloride to collapse the transmembrane pH gradient. The fluorescence intensity was measured using a microplate reader, and the mean values obtained from three independent experiments are shown. AU, arbitrary units. **(B)** Calibration curves for wild-type BGR1 and BGS2 (BGR1 *tofI*::Ω) for measuring internal pH. The data for the calibration curves were obtained as ratios (R_370/470_), and the curves were used to determine internal pH. **(C)** External pH, R_370/470_, and internal pH of wild-type BGR1 and BGS2 (BGR1 *tofI*::Ω) after 14h in LB broth at 37°C. Data are mean±SE of triplicate experiments.

**Figure 8:**
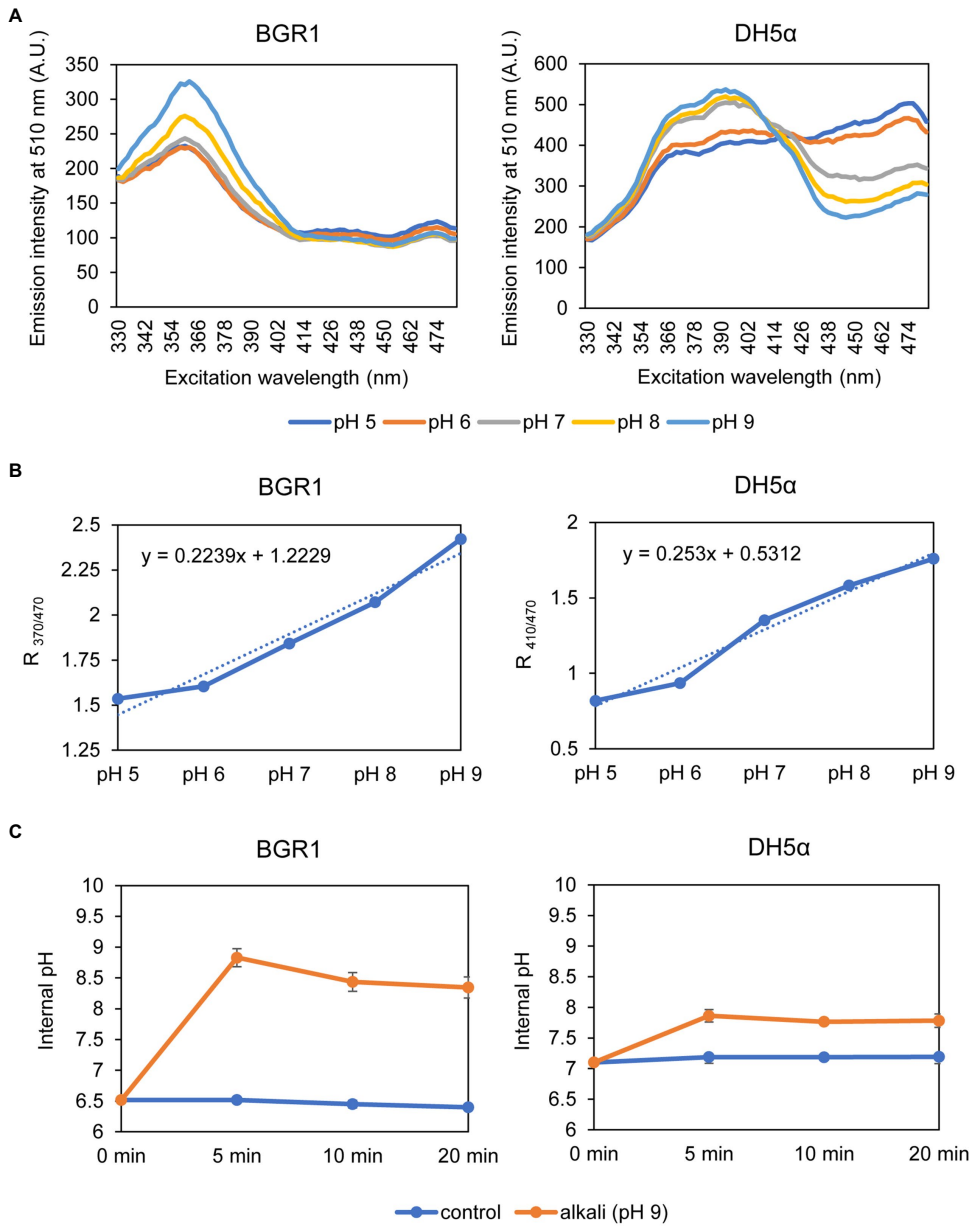
Measurement of the internal pH of wild-type BGR1 and *E. coli* DH5α. **(A)** Fluorescence excitation spectrum of pHluorin for wild-type BGR1 and *E. coli* DH5α (330–482nm; emission, 510nm). Wild-type BGR1 and *E. coli* DH5α were grown in LB broth for 14h at 37°C, and the cells were resuspended in DPBS at different pH (pH 5, 6, 7, 8, or 9) in the presence of 40mM potassium benzoate and 40mM methylamine hydrochloride. The fluorescence intensity was measured using a microplate reader, and the mean values obtained from three independent experiments are shown. AU, arbitrary units. **(B)** Calibration curves for wild-type BGR1 and *E. coli* DH5α for measuring internal pH. The data for the calibration curves were obtained as the ratios between the excitation peak (R_370/470_ or R_410/470_), and the curves were used to determine the internal pH. **(C)** Changes in internal pH values after exposure to alkaline stress at pH 9 in DPBS in wild-type BGR1 and *E. coli* DH5α. Data are mean±SE of triplicate experiments.

Because alkaline stress-induced cell death was correlated with membrane depolarization, cellular pH in *B. glumae* was examined under alkaline environmental conditions using the pH-sensitive ratiometric probe pHluorin. The fluorescent excitation spectra following emission at 510nm showed clear differences across various pH ranges between wild-type BGR1 and BGS2 (BGR1 *tofI*::Ω; Figure 7A). The standard calibration curve and internal pH for wild-type BGR1 and BGS2 (BGR1 *tofI*::Ω) were obtained by calculating R_370/470_ values (Figure 7B). The external pH for wild-type BGR1 and BGS2 (BGR1 *tofI*::Ω) was 5.2 and 8.5, respectively, as the cultures entered the stationary phase (Figure 7C). In wild-type BGR1, the internal pH was maintained at 6.6 even though the external pH of the culture fluid was acidic at pH 5.2, whereas the internal pH of BGS2 (BGR1 *tofI*::Ω) reached 8.8 (Figure 7C). Using the same method, we determined the internal pH of *B. glumae* BGR1 and *E. coli* DH5α following exposure to pH 9 (Figure 8). The fluorescent excitation spectra following emission at 510nm were obtained, and the standard calibration curve was calculated for measuring internal pH (Figures 8A,B). The internal pH of *B. glumae*, which was maintained at pH 6.5 before exposure to pH 9, rapidly increased to 8.8 after exposure to pH 9 and did not recover, whereas the internal pH of *E. coli* remained under 8 and did not fluctuate significantly after the shift to pH 9 (Figure 8C).

**Figure 9:**
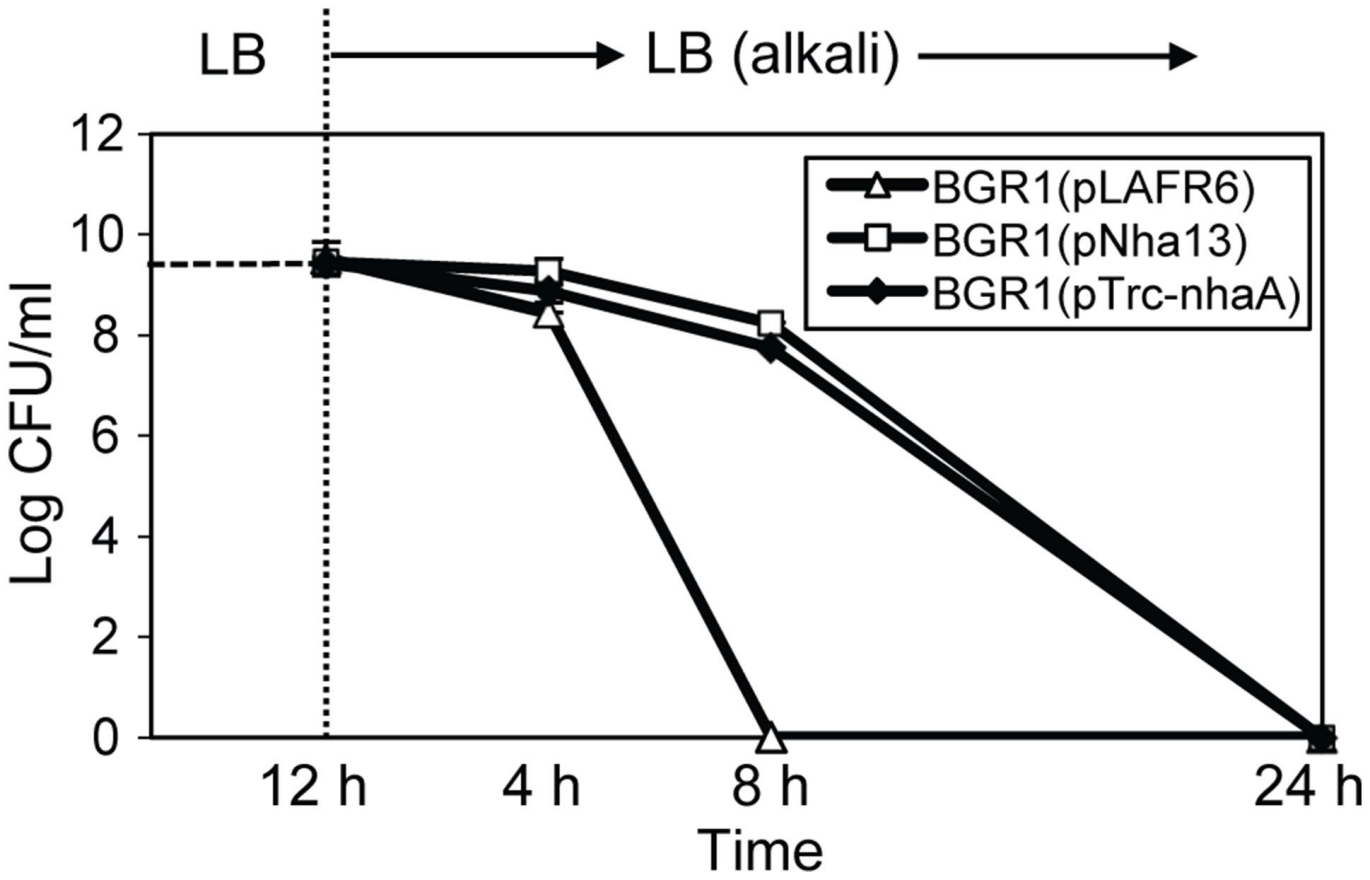
Cell viability of wild-type BGR1 carrying pLAFR6, pNha13, or pTrc-nhaA after exposure to alkaline stress at pH 9. pNha13 and pTrc-nhaA indicate pLAFR6 containing the *nhaA* gene derived from *E. coli* with its native promoter or the *nhaA* gene with the *trc* promoter, respectively. The cell population was determined based on the number of CFUs using plate-counting methods. Data are mean±SE of triplicate experiments.

Because the maintenance of a homeostatic neutral cellular pH in alkali-tolerant bacteria generally relies on Na^+^/H^+^ antiporter function, we tested two scenarios. First, we tested whether the constitutive expression of genes annotated as Na^+^/H^+^ antiporters alleviated alkaline toxicity in *B. glumae*. Second, we heterologously expressed genes involved in maintaining cellular pH homeostasis in other bacteria to determine whether their heterologous expression would affect the viability of *B. glumae* under toxic alkaline conditions. Four putative Na^+^/H^+^ antiporter genes in *B. glumae* exhibited low homology with Na^+^/H^+^ antiporter genes of known alkali-resistant bacteria (Supplementary Table S4). The constitutive expression of each gene in wild-type BGR1 under the *trc* promoter did not rescue alkali toxicity (Supplementary Table S4). On the other hand, when the Na^+^/H^+^ antiporter gene *nhaA* derived from *E. coli* was expressed from its native promoter in *B. glumae*, the population density of wild-type BGR1 with the *nhaA* gene in pLAFR6, named pNha13, declined less during 8h of exposure to pH 9 compared to the density of wild-type BGR1 with the empty vector pLAFR6 (Figure 9). When *nhaA* was constitutively expressed with the *trc* promoter, the population density of wild-type BGR1 carrying pTrc-nhaA indicated similar viability to wild-type BGR1 carrying pNha13; however, both could not completely overcome the alkaline toxicity, and cell death was observed after 24h of alkaline stress (Figure 9).

## Discussion

Diverse apoptosis-like cell death phenomena have been reported in bacteria upon exposure to ROS and antibiotics (Chandra et al., 2000; Dwyer et al., 2012). In addition to these stimuli, we showed that alkaline stress causes similar apoptosis-like cell death in the bacterium *B. glumae*, which is inherently sensitive to alkaline conditions. Although the mechanisms underlying resistance to alkaline environments are well known (Padan et al., 2005), there has been little discussion of alkaline stress-induced cell death. In this study, we suggest alkaline stress as a stress factor that induces apoptosis-like cell death and revealed the molecular and physiological mechanisms underlying alkali-induced apoptosis-like cell death in *B. glumae*.

Apoptosis-like cell death is specifically characterized by the degradation of nucleic acids (Wyllie, 1980; Dwyer et al., 2012). DNA damage can be induced by ROS, which are an ordinary side effect of aerobic respiration produced through consecutive single-electron reductions (Cooke et al., 2003). The sustained production of high levels of ROS is considered a potent inducer of apoptosis-like cell death (Jacobson, 1996; Chandra et al., 2000; Simon et al., 2000). Bactericidal antibiotics generate ROS that cause drug-induced death in *E. coli* (Dwyer et al., 2007; Kohanski et al., 2007, 2008), and some antibiotics cause bacterial cells to undergo apoptosis-like cell death through the generation of intracellular ROS accompanied by DNA degradation (Dwyer et al., 2012). Our study demonstrated that both ROS generation and the upregulation of nucleotide-degrading enzymes occurred in QS mutants and wild-type BGR1 following exposure to alkaline stress. RNA degradation mediated by elevated ribonuclease expression is considered another feature of apoptosis-like cell death in some bacteria (Erental et al., 2014). The elevated expression of Hu-beta under alkaline stress may play a role in the disintegration of chromosomal DNA. However, the putative transcriptional regulators responsible for the upregulation of three genes involved in the degradation of nucleic acids (HNH endonuclease, oligoribonuclease, and ribonuclease E) and the gene encoding the chromosomal binding protein Hu-beta have not been previously identified and will require further investigation.

Studies have shown that membrane depolarization leads to ROS production (Zamzami et al., 1995; Chatterjee et al., 2012). Although ROS accumulation and membrane depolarization under alkaline stress were observed in both the QS mutants and wild-type BGR1, it is not clear whether these phenotypes occur in an ordered manner or simultaneously. Based on the results of this study, it is difficult to conclusively establish whether membrane depolarization is the cause or result of ROS accumulation. Because nigericin caused apoptosis-like cell death in *B. glumae*, it is clear that membrane depolarization was one factor leading to apoptosis-like cell death in *B. glumae* following alkaline stress. Membrane depolarization is an early event of apoptosis-like cell death in other bacteria; it is also thought to cause apoptosis in eukaryotic cells (Hockenbery et al., 1993; Zamzami et al., 1995; Li et al., 1997; Erental et al., 2012, 2014). The relationship between membrane depolarization and the increased expression of genes encoding nucleases was not correlative except for that involving HNH endonuclease. However, increased levels of ROS may cause the upregulation of genes encoding nucleotide-degradation enzymes. Such upregulation of endonucleases during apoptosis with extensive chromosomal cleavage and major nuclear morphology changes has been reported in other bacteria (Arends et al., 1990; Nagamalleswari et al., 2017). Therefore, we presume that alkaline stress-induced cell death of *B. glumae* is the combined result of several mechanisms including membrane depolarization, ROS production, and increased nuclease-encoding gene expression.

Na^+^/H^+^ antiporter genes are critical for maintaining cellular pH homeostasis within neutral pH ranges in Enterobacteriaceae or alkaliphiles as they commonly catalyze H^+^ exchange of Na^+^ across the membrane and consequently regulate the internal pH of the cytoplasm (Ito et al., 1997; Utsugi et al., 1998; Herz et al., 2003). *Escherichia coli* has two antiporter-encoding genes, *nhaA* and *nhaB*, that specifically exchange Na^+^ for H^+^ (Bakker, 1992). *nhaA* is indispensable for adaptation to high salinity and growth at alkaline pH (Taglicht et al., 1993). The other Na^+^/H^+^ antiporter genes that confer resistance against alkali toxicity, *nhaC* and *nhaD*, have been found in alkaliphiles and *Vibrio* spp., respectively (Ito et al., 1997; Herz et al., 2003). A unique Na^+^/H^+^ antiporter gene, *nhaP*, which is not homologous to *nhaA*–*D*, was found in *Pseudomonas aeruginosa* (Utsugi et al., 1998). Because the putative antiporter genes found in *B. glumae* do not exhibit significant homology with the aforementioned Na^+^/H^+^ antiporter genes, this may explain the intrinsic sensitivity of *B. glumae* to alkaline pH. Heterologous expression of *nhaA* from *E. coli* in *B. glumae* somewhat rescued the sensitivity to alkaline toxicity, which strongly suggests that *B. glumae* does not have Na^+^/H^+^ antiporter genes that facilitate survival at high environmental pH. The alkaline tolerance from heterologous gene expression suggests that *B. glumae* has its own mechanism by which it can express *nhaA* even with the native promoter of *E. coli* in response to alkaline pH. In this study, we only investigated heterologous expression of the *nhaA* gene, which encodes the major Na^+^/H^+^ antiporter of *E. coli*, in *B. glumae*. However, it will be necessary to further examine whether greater alkali resistance can be induced by introducing *nhaA* and *nhaB* from *E. coli* in combination, or *nhaC*, *nhaD*, and *nhaP* from alkaliphiles or *P. aeruginosa*.

From an evolutionary perspective, the types of genes present in the genome of *B. glumae* point to it being a plant pathogen. As such, it is important to note that the ability of the bacterium to colonize the apoplast is critical for establishing a parasitic lifestyle (Pfeilmeier et al., 2016). Considering the slightly acidic conditions of the apoplast, we can infer that some genes involved in alkaline tolerance might have been lost in *B. glumae* as an evolutionary adaptive mechanism. It would be interesting to explore whether such evolutionary traits can be found in other plant-associated bacteria. The predicted responses of *B. glumae* to different environmental pH based on their evolved characteristics are in contrast to those of *E. coli* cells, which are part of the intestinal microflora in animals and humans and are able to grow at pH levels of 4.5–9 (Slonczewski et al., 1981; De Jonge et al., 2003). With improved understanding of the growth and survival of *B. glumae* under alkaline conditions, seed-borne infection can be prevented *via* alkaline treatment of seeds or areas of rice and other field crops suspected to be at risk of infection can undergo preventative local treatment.

## Data Availability Statement

The original contributions presented in the study are included in the article/**Supplementary Material**, further inquiries can be directed to the corresponding authors.

## Author Contributions

YN, YK, and IH designed the experiments, contributed to reagents, materials, and analysis tools, and wrote the paper. YN, YK, and EG performed the experiments. YN, YK, EG, and IH analyzed the data. All authors contributed to the article and approved the submitted version.

## Funding

This research was supported by a National Research Foundation of Korea (NRF) funded by the Science and Technology (no. 500–20170088) and Ministry of Education (no. 2020R1I1A1A01070551) of the Korean government.

## Conflict of Interest

The authors declare that the research was conducted in the absence of any commercial or financial relationships that could be construed as a potential conflict of interest.

## Publisher’s Note

All claims expressed in this article are solely those of the authors and do not necessarily represent those of their affiliated organizations, or those of the publisher, the editors and the reviewers. Any product that may be evaluated in this article, or claim that may be made by its manufacturer, is not guaranteed or endorsed by the publisher.
