# Supplementary material for: Membrane Depolarization and Apoptosis-Like Cell Death in an Alkaline Environment in the Rice Pathogen Burkholderia glumae
Source: Front Microbiol. 2021 Oct 12;12:755596. doi: 10.3389/fmicb.2021.755596 (PMC8546246; doi:10.3389/fmicb.2021.755596)
Supplement: Supplementary file 1 [file Data_Sheet_1.pdf]

Supplementary Material

Figure S1. Quantification of GFP-tagged Hu-beta and HNH endonuclease using a PerkinElmer Victor X3 microplate reader. **(A)** GFP fluorescence from tagged Hu-beta and HNH endonuclease in wild-type BGR1 and QS mutants was measured after 18 h at 37°C. **(B)** The fluorescence intensity of GFP-tagged HNH endonuclease and Hu-beta in wild-type BGR1 was detected after 4 h of artificially induced alkaline stress. Data are mean ± SE of triplicate experiments. The letters (a, and b) above each mean represent groupings of statistical significance based on ANOVA/Tukey's correction for multiple comparisons. A value of $p < 0.05$ represents significant differences among strains.

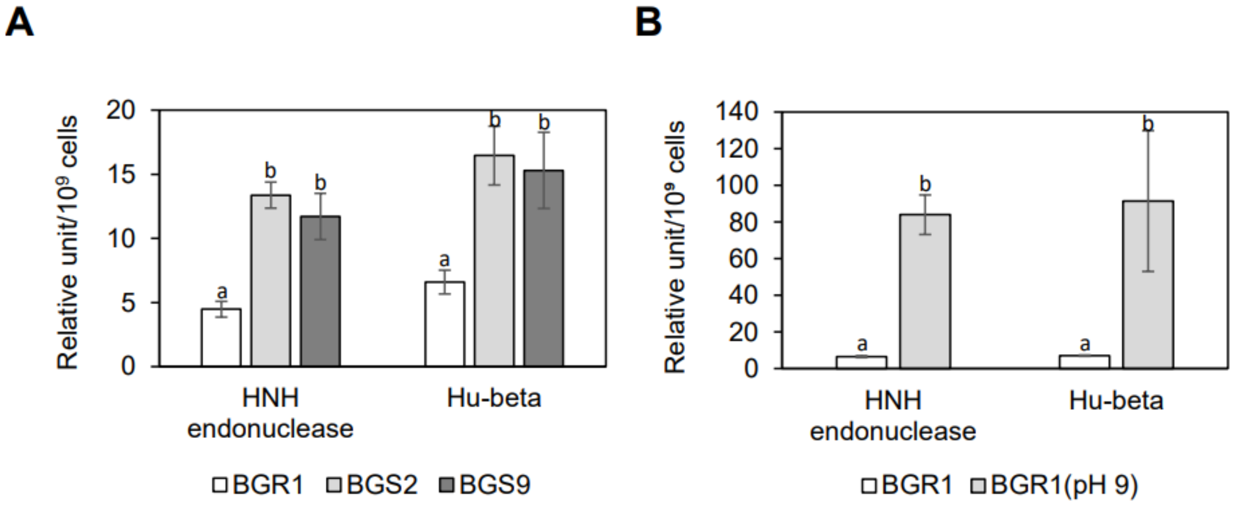

Table S1. Bacterial strains and plasmids used in this study.

Strain or plasmid	Genotype or phenotype[a]	Reference or source
Escherichia coli		
DH5α	F^-Φ80d*lac*ZΔ*M15*Δ(*lac*ZYA-*argF*) *U169endA1 recA1hsd1hsdR17*($r_k^- m_k^+$) *deoRthi*-1*supE44*λ^- *gyrA96 relA1*	Gibco BRL
DH5α λ*pir*	F^-Φ80d*lac*ZΔ*M15*Δ(*lac*ZYA-*argF*) *U169endA1 recA1hsd1hsdR17*($r_k^- m_k^+$) *deoRthi*-1*supE44*λ^- *gyrA96 relA1*, λ*pir*	(Choi et al., 2006)
HB101	F^-*mcrBmrrhsdS20*($r_B^- m_B^-$)*recA13leuB6ara-14 proA2lacY1galK2xyl-5 mtl-1 rpsL20*(Sm^r) *supE44*λ^-	Gibco BRL
S17-1 λ*pir*	Tp^r Sm^r *recA*, *thi*, *pro*, *hsdR*$^-$*M*$^+$ RP4::2-Tc::Mu:Km ::Tn*7*, λ*pir*	(Choi et al., 2006)
Burkholderia glumae		
BGR1	wild type, Rif^r	(Jeong et al., 2003)
BGS2	BGR1 *tofI*::Ω	(Kim et al., 2004)
BGS9	BGR1 *qsmR*::Ω	(Kim et al., 2007)
BGHU	BGR1::Hu-beta-eGFP-miniTn*7*	This study
S2HU	BGS2::Hu-beta-eGFP-miniTn*7*	This study
S9HU	BGS9::Hu-beta-eGFP-miniTn*7*	This study
BGHNH	BGR1::HNH endonuclease-eGFP-miniTn*7*	This study
S2HNH	BGS2:: HNH endonuclease -eGFP-miniTn*7*	This study
S9HNH	BGS9:: HNH endonuclease -eGFP-miniTn*7*	This study
Plasmids		
pBluescript II SK(+)	Cloning vehicle; phagemid, pUC derivative, Amp^r	Stratagene
pRK2013	Tra^+, ColE1 replicon, Km^r	(Keen et al., 1988)
pUC18R6K-miniTn*7*T-Tc	Mobilizable mini-Tn*7*-Tc vector, Amp^r, Tet^r	(Choi et al., 2006)
pTNS2	Plasmid expressing *tnsABCD* from P_{lac}, Amp^r	(Kim et al., 2013)

pJW23	Coding region of *egfp* was cloned into pGEM-T Easy vector	(Jang et al., 2014)
pLAFR3	Tra$^-$, Mob$^+$ RK2 replicon, Tet^r	(Staskawicz et al., 1987)
pLAFR6	pLAFR3 but without *lacZα*, contains multilinker of pUC18 flanked by synthetic *trp* terminators, Tet^r	(Huynh et al., 1989)
pBP1	*trc* promoter and *Nde*I site were cloned upstream of MSC in pBluescript II SK(+)	This study
pHU1	499-bp PCR product containing the promoter and coding region of Hu-beta inserted at the *Sma*I site of pBluscript II SK(+)	This study
pHNH1	666-bp PCR product containing the promoter and coding region of HNH endonuclease inserted at the *Sma*I site of pBluscript II SK(+)	This study
pHU2	0.5-kb *Sac*I–*Xho*I DNA fragment harboring Hu-beta from pHU1 cloned into pJW23	This study
pHNH2	0.6-kb *Sac*I–*Xho*I DNA fragment harboring HNH endonuclease from pHNH1 cloned into pJW23	This study
pHU3	1.3-kb *Sac*I–*Eco*RI DNA fragment harboring Hu-beta–*egfp* from pHU2 cloned into pUC18R6K-miniTn*7*T-Tc	This study
pHNH3	1.4-kb *Sac*I–*Eco*RI DNA fragment harboring HNH endonuclease–*egfp* from pHNH2 cloned into pUC18R6K-miniTn*7*T-Tc	This study
pPHI1	717-bp PCR product of ratiometric pHluorin inserted at the *Sma*I site of pBluscript II SK(+)	This study
pPHI2	0.5-kb *Nde*I–*Bam*HI partial DNA fragment harboring ratiometric pHluorin cloned into pBP1 carrying *trc* promoter	This study
pPHI3	0.2-kb *Nde*I–*Nde*I partial DNA fragment harboring ratiometric pHluorin cloned into pPHI2 carrying *trc* promoter	This study

pPHI4	1.2-kb *Sac*I–*Bam*HI DNA fragment containing *trc* promoter and harboring ratiometric pHluorin from pPHI3 cloned into pLAFR6	This study
pNha1	1.6-kb PCR product of Na^+/H^+ antiporter (BGLU_1G32530) inserted at the *Sma*I site of pBluscript II SK(+)	This study
pNha4	1.6-kb *Nde*I–*Bam*HI DNA fragment harboring Na^+/H^+ antiporter (BGLU_1G32530) cloned into pBP1 carrying *trc* promoter	This study
pNha7	1.6-kb *Sac*I–*Kpn*I DNA fragment containing *trc* promoter and harboring Na^+/H^+ antiporter (BGLU_1G32530) from pNha4 cloned into pLAFR6	This study
pNha2	1.6-kb PCR product of Na^+/H^+ antiporter NhaD (BGLU_1G09320) inserted at the *Sma*I site of pBluscript II SK(+)	This study
pNha5	1.6-kb *Nde*I–*Bam*HI DNA fragment harboring Na^+/H^+ antiporter NhaD (BGLU_1G09320) cloned into pBP1 carrying *trc* promoter	This study
pNha8	1.6-kb *Sac*I–*Kpn*I DNA fragment containing *trc* promoter and harboring Na^+/H^+ antiporter NhaD (BGLU_1G09320) from pNha5 cloned into pLAFR6	This study
pNha20	1.3-kb PCR product of Na^+/H^+ antiporter-like protein (BGLU_2G00450) inserted at the *Sma*I site of pBluscript II SK(+)	This study
pNha22	1.3-kb *Nde*I–*Bam*HI DNA fragment harboring Na^+/H^+ antiporter-like protein (BGLU_2G00450) cloned into pBP1 carrying *trc* promoter	This study
pNha24	1.3-kb *Sac*I–*Bam*HI DNA fragment containing *trc* promoter and harboring Na^+/H^+ antiporter-like protein (BGLU_2G00450) from pNha22 cloned into pLAFR6	This study

pNha21	1.6-kb PCR product of Na^+/H^+ antiporter (BGLU_2G17030) inserted at the *Sma*I site of pBluscript II SK(+)	This study
pNha23	1.6-kb *Nde*I–*Bam*HI DNA fragment harboring Na^+/H^+ antiporter (BGLU_2G17030) cloned into pBP1 carrying *trc* promoter	This study
pNha25	1.6-kb *Xba*I–*Hind*III DNA fragment containing *trc* promoter and harboring Na^+/H^+ antiporter (BGLU_2G17030) from pNha23 cloned into pLAFR6	This study
pNha12	1.3-kb PCR product of native promoter and *nhaA* of *E. coli* inserted at the *Sma*I site of pBluscript II SK(+)	This study
pNha13	1.3-kb *Sac*I–*Kpn*I DNA fragment harboring native promoter and *nhaA* of *E. coli* cloned into pLAFR6	This study
pTrc-nhaA	1.3-kb *Sac*I–*Hin*III DNA fragment harboring *trc* promoter and *nhaA* of *E. coli* cloned into pLAFR6	This study

Rif^r, rifampicin resistance; Tet^r, tetracycline resistance; Km^r, kanamycin resistance; Amp^r; ampicillin resistance; Sm^r, streptomycin resistance.

Table S2. Primers used for PCR and qRT-PCR in this study. All primers used were purchased from Macrogen (Seoul, Korea).

Primers	Sequence (5' to 3')
1g08730-FS	CGAACTGAACAACGACTG GA
1g08730-FL	AGCTGCAGGACACGTTCG
1g08730-R	GCATCTCCTTCAGGTTCAGC
1g15690-F	GTCTACGCTCACCGAATTGC
1g15690-R	GACAGTATTGGCCGCGTTCT
1g09120-F	CACAGATCGAGGCGTTCCT
1g09120-R	GGTAATGGAAGAAGCGCTCG
1g09400-F	ATACCGAATCGTCGGCGCTG
1g09400-R	TCCTGGCGCTTTTCGTTGAG
1g13530-F	CGAAGCGGATTTATCGAAAG
1g13530-R	ATTGCGCGCGATCCTCTCAC
16S rRNA-F	TCTGAGAGGACGACCAGCCA
16S rRNA-R	CGAAGGCCTTCTTCACACAC
PHu-F	CAGGAGCTCATCCGAACCCTTTTCGGCAC
PHu-R	ACCCTCGAGGTTTAAGGCATCTTTCAGTGCTTTAC
PyajD-F	CAGGAGCTCCGCCAATTTCGATTTCGGTGGCCA
PyajD-R	ACCCTCGAGGTCGTCTCGCGAGTGTGTGAGC
glmS-down	AGCCGCAGATCATCGCCTG
1g32800-up	CCACGCATCGAAATCCTC
pHluorin-F	CCATATGAGTAAAGGAGAAGAACTTTTCACTGG
pHluorin-R	CCGGATCCTTATTTGTATAGTTCATCCATGCC
NhaA-F	CCAAGAGCTCCTATCTGCCGTTCAGCTAATGC
NhaA-R	ACCGTGGGCCCCGTGTCA
1g32530-F	CCAACATATGGAAATCGTCTTCACCGT
1g32530-R	GGTTGGATCCTCAGACGAGCCCTTTCTTG
1g09320-F	CCAACATATGACGGCCGTCACGCGGCGCCAGGCCGCTTTCCGTTTG

1g09320-R	GGTTGGATCCGTTCAGGCGGAAAAGAACAG
2g00450-F	ACCCATATGCTGCATGAAACCGAGTGG
2g00450-R	TTGGGATCCTCAGCGGCTCGAGCG
2g17030-F	ACCCATATGTCCGCCGTGTCCGTCTTC
2g17030-R	TTGGGATCCTCATTCCATGGCGTGGCG

F- forward primer; R- reverse primer

Table S3. Results of RNA sequencing analysis of genes encoding nucleic acid-degrading enzymes and chromosomal binding HU-beta in *B. glumae*.

Gene ID[a]	Gene	Reads per kilobase per million mapped reads (RPKM)					
		6 h			10 h		
		BGR1	BGS2	BGS9	BGR1	BGS2	BGS9
BGLU_1G15690	*yajD*	42.95645	97.42078	48.58727	41.75269	115.0284	75.58977
BGLU_1G09120	*orn*	216.0408	267.4828	208.2017	75.27826	236.8183	211.9989
BGLU_1G09400	*rne*	338.5968	400.9831	322.6752	97.6271	263.9991	262.0803
BGLU_1G13530	*hupB*	28.4161	559.7489	128.4933	43.33637	581.4282	287.1304

[a]Gene IDs were obtained from the *B. glumae* BGR1 genome database (GenBank accession numbers: CP001503–CP001508).

The RNA sequencing data were deposited in the Gene Expression Omnibus database (www.ncbi.nlm.nih.gov/geo) under accession number GSE36485.

Table S4. Homology between known cation-proton antiporter genes in other bacteria and putative Na^+/H^+ antiporter genes in *B. glumae* and effect against alkaline toxicity.

Gene ID[a]	Homology[b]	Homology with known cation-proton antiporter[c] (identity/positive rate)	Effectiveness against alkaline toxicity[d]
BGLU_1G32530	*Pseudomonas acidophila* NhaP-type Na^+/H^+ and K^+/H^+ antiporter (70.27%)	NhaP (21%/35%)	–
BGLU_1G09320	*Pseudomonas acidophila* Na^+/H^+ antiporter (69.91%)	NhaB (19%/34%)	–
BGLU_2G00450	*Chthoniobacterales bacterium* Na^+/H^+ antiporter (60.74%)	NhaP (19%/31%)	–
BGLU_2G17030	*Pseudomonas acidophila* NhaP-type Na^+/H^+ and K^+/H^+ antiporter (72.35%)	NhaP (21%/35%)	–

[a]Gene IDs were obtained from the *B. glumae* BGR1 genome database (GenBank accession numbers: CP001503–CP001508).

[b]Numbers in parentheses refer to the percentage of similarity.

[c]Homology with *Escherichia coli* NhaA, NhaB, and ChaA; *Vibrio cholerae* NhaC and NhaD; and *Pseudomonas aeruginosa* NhaP.

[d]Effectiveness of putative Na^+/H^+ antiporter genes expressed under the *trc* promoter in *B. glumae* BGR1 against alkaline stress. The minus (–) signs denote that the expression of putative Na^+/H^+ antiporter genes under the *trc* promoter did not significantly affect cell viability in *B. glumae* BGR1 suffering from alkaline stress at pH 9.

SUPPLEMENTAL REFERENCES

Choi, K.-H., Deshazer, D., and Schweizer, H.P. (2006). mini-Tn 7 insertion in bacteria with multiple glmS-linked att Tn 7 sites: example *Burkholderia mallei* ATCC 23344. *Nat. Protoc*. 1, 162. doi: 10.1038/nprot.2006.25

Huynh, T.V., Dahlbeck, D., and Staskawicz, B.J. (1989). Bacterial blight of soybean: regulation of a pathogen gene determining host cultivar specificity. *Science*. 245, 1374-1377. doi: 10.1126/science.2781284

Jang, M.S., Goo, E., An, J.H., Kim, J., and Hwang, I. (2014). Quorum sensing controls flagellar morphogenesis in *Burkholderia glumae*. *PLoS One*. 9, e84831. doi: 10.1371/journal.pone.0084831

Jeong, Y., Kim, J., Kim, S., Kang, Y., Nagamatsu, T., and Hwang, I. (2003). Toxoflavin produced by *Burkholderia glumae* causing rice grain rot is responsible for inducing bacterial wilt in many field crops. *Plant Dis*. 87, 890-895. doi: 10.1094/PDIS.2003.87.8.890

Keen, N.T., Tamaki, S., Kobayashi, D., and Trollinger, D. (1988). Improved broad-host-range plasmids for DNA cloning in gram-negative bacteria. *Gene*. 70, 191-197. doi: 10.1016/0378-1119(88)90117-5

Kim, J., Heindl, J.E., and Fuqua, C. (2013). Coordination of division and development influences complex multicellular behavior in *Agrobacterium tumefaciens*. *PLoS One*. 8, e56682. doi: 10.1371/journal.pone.0056682

Kim, J., Kang, Y., Choi, O., Jeong, Y., Jeong, J.E., Lim, J.Y., et al. (2007). Regulation of polar flagellum genes is mediated by quorum sensing and FlhDC in *Burkholderia glumae*. *Mol. Microbiol*. 64, 165-179. doi: 10.1111/j.1365-2958.2007.05646.x

Kim, J., Kim, J.G., Kang, Y., Jang, J.Y., Jog, G.J., Lim, J.Y., et al. (2004). Quorum sensing and the LysR-type transcriptional activator ToxR regulate toxoflavin biosynthesis and transport in *Burkholderia glumae*. *Mol. Microbiol*. 54, 921-934. doi: 10.1111/j.1365-2958.2004.04338.x

Staskawicz, B., Dahlbeck, D., Keen, N., and Napoli, C. (1987). Molecular characterization of cloned avirulence genes from race 0 and race 1 of *Pseudomonas syringae* pv. *glycinea*. *J. Bacteriol*. 169, 5789-5794. doi: 10.1128/jb.169.12.5789-5794.1987
